# Emotional dysregulation and health related quality of life in young adults with ADHD: a cross sectional study

**DOI:** 10.1186/s12955-021-01904-8

**Published:** 2021-12-20

**Authors:** Maayan Ben-Dor Cohen, Eran Eldar, Adina Maeir, Mor Nahum

**Affiliations:** 1 School of Occupational Therapy, Faculty of Medicine, Hebrew University of Jerusalem, Jerusalem, Israel; 2 Psychology Department, Faculty of Social Sciences, Hebrew University of Jerusalem, Jerusalem, Israel; 3 Department of Cognitive Sciences, The Hebrew University of Jerusalem, Jerusalem, Israel

**Keywords:** ADHD, Emotion dysregulation, Health related quality of life, Young adults

## Abstract

**Objective:**

Attention Deficit Hyperactivity Disorder (ADHD) is associated with emotional dysregulation (ED) and impaired health related quality of life (HRQoL). However, the role of ED in explaining the relationship between ADHD and HRQoL is unclear. The purpose of the present study was to do so in a sample of non-referred young adults with and without ADHD.

**Method:**

The study design was cross-sectional. A non-clinical sample of 63 young adults with ADHD (mean age = 24.86 years, SD = 3.25, 78% university students) and 69 gender-matched controls (mean age = 23.84 years, SD = 2.59, 89% university students) were recruited. The Adult ADHD Quality-of-Life scale was used to measure HRQoL; The Self-Report Wender–Reimherr Adult Attention Deficit Disorder Scale and the Difficulties in Emotion Regulation Scale were used to measure ED. Group differences on all measures were tested using univariate and multivariate analyses of covariance, while controlling for age. Finally, a moderation analysis was used in order to examine the impact of ED on HRQoL beyond that accounted for by ADHD symptoms.

**Results:**

Both HRQoL and ED were significantly worse for the ADHD group compared to the control group. The medication status of the ADHD group participants had no significant effect on the level of ADHD symptoms, ED or HRQoL. ED moderated the effect of ADHD symptoms on HRQoL for the ADHD group.

**Conclusion:**

The findings support the centrality of ED in ADHD and its crucial influence on HRQoL. Young adults with ADHD and high levels of ED are at risk for aversive impact on their well-being regardless of their ADHD symptoms level.

## Plain English summary

Young adults with Attention Deficit Hyperactivity Disorder (ADHD) struggle with poor quality of life (QoL). Emotional regulation is one’s ability to modify their emotional state to promote adaptive, goal-oriented behaviors. Emotional dysregulation is a common yet neglected feature of people with ADHD. Our results show that young adults with ADHD are twice more likely to suffer from emotional dysregulation then their peers. Moreover, higher levels of emotional dysregulation predicted lower levels of QoL. These findings support the centrality of ED in ADHD and their crucial influence on everyday QoL. These findings are important not only on the theoretical level, but may also contribute to the design of interventions that aim to promote quality of life.

## Background

Quality of Life is a multidimensional concept that reflects one's subjective perception of his/her position in life in relation to their goals, expectations, standards, and concerns [1, 2]. Health related Quality of Life (HRQoL) represents the impact of a health condition on quality of life [3]. Different health conditions could negatively affect one's ability to live a fulfilling life, as a consequence of specific symptoms, impairments, and changes in functional state [4]. At the same time, HRQoL is distinct from the symptoms and objective functional outcomes of different health conditions. Thus, any symptom can lead to multiple impairments in different life roles, leading to a synergistic negative impact on daily life. As a result, the overall long-term burden of a chronic health condition is often greater than the simple sum of individual functional impairments [5]. This places HRQoL as a significantly important clinical outcome in the assessment of the impact of health conditions on an individual's meaningful occupations and well-being [2, 6–11].

Attention Deficit Hyperactivity Disorder (ADHD) is a neurodevelopmental disorder, characterized by core symptoms of inattention, impulsivity, and hyperactivity [12]. The worldwide prevalence of ADHD in young adults is estimated to be 5% [13]. HRQoL has been shown to be significantly compromised in the heterogenic population of adults with ADHD [5, 10, 14, 15]. Specifically, previous studies that examined HRQoL in college students with and without an ADHD diagnosis found that young adults with ADHD reported low HRQoL compared to their non-ADHD peers [6, 16]. In addition, ADHD symptom severity and functional impairment have been shown to predict lower HRQoL in adults with ADHD [17–22]. Yet, the relatively moderate effect sizes of the associations between ADHD symptoms and HRQoL imply that there may be additional factors contributing to HRQoL in adult ADHD [23, 24]. Specifically, pharmacological and psychosocial treatments, comorbid psychopathology, and drug use did not moderate the relationship between ADHD and HRQoL [16]. Further investigation is therefore needed in order to better understand which ADHD-associated factors significantly impact HRQoL in young adults with ADHD.

Emotion dysregulation (ED), an individual's inability to modify one’s emotional state to promote adaptive, goal-oriented behaviors [25] has been hypothesized as a factor which may impact HRQoL in ADHD [2, 26]. ED is highly prevalent in adults with ADHD, with recent clinic-based studies reporting impaired ED in 32%-86% of adults with ADHD [27–32]. The definition and measurement of ED are in substantial disarray in general, and specifically in ADHD, reflecting different perspectives on the relationship between ED and ADHD [33]. On the one hand, ED was suggested to be an additional core symptom specific to ADHD or at least to a subtype of ADHD with which certain facets of ED are uniquely associated (such as temper control, affective lability, emotional over reactivity, emotional impulsivity and deficient emotional self-regulation) [33–37]. This perspective is supported by the high prevalence of ED in ADHD, regardless of comorbidities [29, 36]. Moreover, ED is associated with ADHD symptom severity and respond to pharmacological and psychotherapeutic treatments in adults, similarly to ADHD core symptoms [31, 38–42]. On the other hand, however, ED is proposed as another dimensional key factor within the Research Domain Criteria Initiative (RDoc) that could distinguish between people with and without many mental disorders [19]. This may imply that ADHD-associated ED is not specific to ADHD; instead, it has transdiagnostic mechanisms and etiology which are shared with other clinical conditions (such as the ability to monitor and accept one’s emotional state, to control impulsive reaction and to use effective strategies in order to engage in goal-directed behaviors) [43]. This ongoing debate regarding the ADHD-specific vs. transdiagnostic nature of ED in ADHD, and the lack of agreed upon measurement approaches, warrants combining different measures to capture both perspectives on ED.

Regardless of the exact definition of ED in the context of ADHD, the presence of ED in ADHD is associated with multiple functional impairments (such as, social participation, marital status, driving, money management and arrests). ED, as measured by ADHD-related instruments, is associated with higher levels of impairment in clinical samples of adults and young adults with ADHD, beyond what could be predicted by ADHD symptoms and comorbidity [27, 36, 44]. Using a general transdiagnostic assessment tool, ED was found to mediate the relationship between ADHD and functional impairment in adults with ADHD [45]. Also, the negative effect of ADHD symptoms level on the quality of romantic relationships was no longer significant when ED was added as a mediator in a sample of college students [46].

Interestingly, only a handful of studies have examined the direct contribution of ED to HRQoL in ADHD, yielding mixed results. Using a clinical sample of adults with ADHD, two studies reported that ED severity was significantly associated with ADHD symptoms severity and with HRQoL, regardless of medication status [44, 47]. On the other hand, in a study which examined the neuropsychological factors in a general population of young adults, ED was not found to be a unique contributor to HRQoL beyond other neuropsychological deficits [48]. No study to date, to the best of our knowledge, has directly examined the impact of ED on HRQoL in a sample of young adults with ADHD beyond that of ADHD symptoms. Understanding the unique impact of ED on HRQoL is expected to deepen our understanding of the highly varied clinical picture of young adults with ADHD and its effects on one's daily living experiences and well-being.

In the current study, we examined the potential moderating effect of ED on the relationship between ADHD symptoms and HRQoL in non-referred young adults with ADHD. We first examined the differences in ED between adults with and without ADHD using two different self-report measures, collectively reflecting both the transdiagnostic and the ADHD-related nature of ED. Next, we examined the moderating role of ED in explaining HRQoL in young adults with ADHD beyond the scope of ADHD symptoms. This study should contribute to understanding of the variance of HRQoL among the heterogenic population of young adults with ADHD.

## Methods

### Study design

The current study is a part of a larger cross-sectional study which involved baseline assessments performed in the lab in addition to 5 days of ecological momentary assessments (EMA) of mood reporting and cognitive testing performed 5 times/day using participant’s mobile phones. Here we report the results of the baseline assessments only. Participants were recruited for the study via online advertisements in student organization groups and via posts in social media. Eligible participants were invited to the Computerized Neurotherapy Laboratory at the Hebrew university where baseline assessments took place. Data was collected by research assistants who were not part of the teaching stuff at the university. All participants signed an informed consent prior to participation in any study-related activities. Participants were compensated for their participation in the study, receiving US$100 for completion of the entire study. The study was approved by the Institutional Review Board (IRB) Ethics Committee of the Hebrew University of Jerusalem (reference ID: 08112017).

### Participants

Over a period of 1 year (from January 2018 to January 2019) 155 young adults were recruited for the study. ADHD group participants were required to present a documented valid medical diagnosis of ADHD given by a certified medial professional (neurologist or psychiatrist). Since information about the diagnostic process was not collected in the study itself, we used the clinical cutoff score of the Adult ADHD Self-Report Scale (ASRS; ≥ 51) to confirm the ADHD diagnosis [49, 50]. Inclusion criterion for the healthy comparison group was an ASRS score below the clinical cutoff. Exclusion criteria for both groups were a diagnosis of a psychiatric or neurological disorder or other major health conditions as determined by self-report. Overall, 23 participants were excluded from the study: 10 from the control group (two due to a reported diagnosis of a psychiatric disorder and eight scored above the clinical cutoff of the ASRS) and 13 from the ADHD group, as they scored below the clinical cutoff of the ASRS. The final sample analyzed included 132 young adults (ages 18–33): 63 adults with ADHD and 69 gender-matched (at the group level) controls. The sample was balanced across the two groups with respect to gender. However, since the ADHD group was slightly older, age was entered as a covariate in group comparison analyses (see Table 1). Medication status for ADHD group participants was recorded and controlled for in data analyses (see Table 2).

**Table 1:** Demographics characteristics of study sample

Characteristics	ADHD (N = 63)	Controls (N = 69)	*t* or ${x}^{2}$	*P* value
	N (%)	N (%)		
*Gender*				
Female	33 (52.4)	38 (55.1)	.096	.757
Male	30 (47.6)	31 (44.9)		
*Age*, M (SD)	24.86 (3.25)	23.84 (2.59)	1.997	.048*
*Education*				
High school graduate	14 (21.2)	4 (5.7)	7.544	.006**
University or post graduate degree	49 (77.8)	66 (94.3)		
*Current productive role*				
Student	35 (55.6)	43 (62.3)	3.550^a^	.299
Working	11 (17.5)	7 (11.5)		
Both	14 (22.2)	18 (26.2)		
Unemployed	3 (4.8)	–		
*Family status*				
Single	38 (60.3)	41 (59.4)	.011	.916
Married/ partnered	25 (39.7)	28 (40.6)		
*ADHD Symptoms*^b^, M (SD)	60.79 (6.35)	39.84 (6.62)	18.524	.000***
*Medication status*				
Daily	14 (22.2)	–		
Irregular use	31 (49.2)	–		
None	18 (28.6)	–		

*M* = mean; *SD* = standard deviation; ^a^Fisher’s Exact Test; ^b^adult ADHD Self Report Scale (ASRS) total score

*p < .05; **p < .01; ***p < .001

**Table 2:** The effect of medication status on outcomes in the ADHD group

	Daily	Irregular use	None	F (2,60)	*p*	η^2^
	M (SD)	M (SD)	M (SD)			
ASRS	60.01 (6.55)	61.68 (6.37)	59.83 (6.33)	.588	.559	.019
WRAADDS ED	19.71 (10.29)	18.81 (10.43)	19.17 (10.56)	.037	.964	.001
DERS	84.07 (24.48)	89.23 (22.85)	89.89 (30.35)	.247	.782	.008
HRQoL	60.24 (16.89)	56.30 (14.15)	60.04 (17.78)	.459	.634	.015

*M* = mean; *SD* = standard deviation; ASRS = adult ADHD Self Report Scale total score; WRAADDS ED = Emotional Dysregulation total score derived from the Wender–Reimherr Adult Attention Deficit Disorder Rating Scale; DERS = Difficulties in Emotion Regulation Scale total score; HRQoL = The adult ADHD quality of life questionnaire total score

*p < .05; **p < .01; ***p < .001

### Measures

ASRS (version 1.1) Symptom Checklist [50] is an instrument designed to measure current ADHD symptoms. The scale consists of 18 items based on the DSM-IV ([51]) criteria for ADHD that are measured on a 5-point scale (0 = never and 4 = very often), yielding a total score which is comprised of the sum of all items and ranging from 0 to 72. A screener score of 51 and over was found to be the most predictive of symptoms consistent with ADHD [49]. In the current study we used the ASRS Hebrew version which has high test–retest reliability (r = 0.60-0.90), a significant discriminant validity and good internal consistency (alpha = 0.82–0.89) [49], which was found also in the current study (alpha = 0.89).

WRAADDS ED, ED severity was assessed using 3 sub-scales from the Self-Report Wender–Reimherr Adult Attention Deficit Disorder Scale (SR-WRAADDS; [34]): temper, affective lability, and emotional over-reactivity. Collectively, the 11 items within the three subscales assess emotional symptoms in adult ADHD. Each item is measured on a 5-point scale (0 = None or slightly, 4 = very much), yielding scores that may range from 0 to 44. A particular area is considered relevant if the sum score of its corresponding subscale is at least equal to the number of items listed in that subscale, resulting ED cutoff achieved with a sum ≥ 11 [52]. The SR-WRAADDS is based on the interviewer-administered Wender Reimberr Adult Attention Deficit Disorder Scale (WRAADDS; [53]). Similar ED scale based on the WRAADDS has been used in several previous studies [29, 30, 38, 39, 54]. The internal consistency reflected the homogeneity of all the WRAADDS subscales, including ED. Cronbach's α for the entire WRAADDS was 0.88. ED indicated the highest internal consistency (Cronbach's α = 0.78) compared to the other WRAADDS subscales (α = 0.65–0.73) [29]. The SR-WRAADDS was translated to Hebrew by our study team with permission from the scale's authors. In the current study the internal consistency (i.e., coefficient α) for the ED subscales ranged from 0.80 to 0.91 and 0.92 for the total score.

The Difficulties in Emotion Regulation Scale (DERS; [43]) was developed to assess ED more comprehensively than existing measures. The DERS 36 items were chosen to reflect difficulties within the following dimensions of emotion regulation: (a) lack of awareness of emotional responses (awareness), (b) lack of clarity of emotional responses (clarity), (c) non-acceptance of emotional responses (non-acceptance), (d) limited access to emotion regulation strategies perceived as effective (strategies), (e) difficulties controlling impulses when experiencing negative emotions (impulse), and (f) difficulties engaging in goal-directed behaviors when experiencing negative emotions (goals). Each item is measured on a 5-point scale (1 = almost never, 5 = almost always), yielding scores that may range from 36 to 180, with higher scores indicating greater difficulties in emotion regulation (i.e., greater ED). The DERS has high internal consistency (*α* = 0.93) [43]. Here we used the validated Hebrew version [55]. The scale's internal consistency for the current study ranged from.70 to 0.89 for the various subscales and 0.95 for the total score.

The Adult ADHD Quality-of-Life scale (AAQoL; [5]) is one of the most commonly used disease-specific instruments to measure HRQoL in adults with ADHD in both research and clinical practice [56, 57]. The AAQoL consists of 29 items rated on a 5-point scale describing frequency of occurrence, yielding a total score and four subscale scores: productivity, life outlook, relationships and psychological health. Total and subscale raw scores are transformed to a 0- to 100-point scale with higher scores indicating better HRQoL. The AAQoL has good internal consistency (Cronbach alpha = 0.93) and good test–retest reliability (ICC alpha = 0.86) and is able to discriminate between groups with and without ADHD [5, 14]. The Hebrew version used here has demonstrated validity in Israeli adults with ADHD [8]. The internal consistency for the current study ranged from 0.74 to 0.90 and 0.94 for the total score.

### Statistical analysis

All analyses were conducted using the IBM SPSS software Version 25.0 [58]. Group differences in demographic variables were examined using chi-square, Fisher's exact tests or independent samples t tests. Univariate analyses of variance (ANOVA) were conducted to investigate the possible effect of medication on the level of ADHD symptoms, ED and HRQoL in the ADHD group. We first assessed the differences between ADHD and controls on WRAADDS ED, DERS and HRQoL total scores using univariate analyses of covariance (ANCOVA), while controlling for age. Next, the differences between groups in ED, DERS and HRQoL subscales were examined using multivariate analyses of covariance (MANCOVA), while controlling for age. Finally, we conducted moderation analysis using PROCESS analysis by Hayes [59]. The analysis utilized a bootstrapping approach, and significance was determined at 95% bias-corrected confidence intervals. All variables were continuous and centered prior to analysis, and the estimated effects reported were unstandardized regression coefficients [59]. The distributions of all study variables met normality criteria of Kolmogorov Smirnoff test. The significance level for general hypothesis testing was set at α = 0.05.

## Results

### Participants' characteristics

A total of 155 participants were considered for eligibility, but the final study sample therefore included a total of 132 participants: 63 in the ADHD group and 69 participants in the healthy comparison group.

The demographic characteristics of the study sample are shown in Table 1. Gender, current productive role and family status were comparable in the two groups. However, the average age in the ADHD group was slightly higher than that of the comparison group (24.86 ± 3.25 vs. 23.84 ± 2.59 for ADHD and comparison groups, respectively; *p* = 0.048). As expected, the ED scores were significantly worse for the ADHD group, and participants in the ADHD group were less educated. The medication status of the ADHD group participants had no significant effect on the level of ADHD symptoms, ED or HRQoL (see Table 2).

### ADHD, ED and HRQoL

Total scores for the WRAADDS ED, DERS and HRQoL in the two groups are presented in Table 3. A one-way ANCOVA, controlling for age, showed significantly higher (i.e., worse) scores in WRAADDS ED and in DERS and significantly lower (i.e., worse) scores in the HRQoL in the ADHD compared with the comparison group. Specifically, 73% of ADHD group participants scored above WRAADDS ED cutoff (a total score sum ≥ 11) compared to 37.7% in the control group [${x}^{2}\left(1, N=63\right)=16.584,p=.000]$. To further investigate where the differences lie, one-way MANCOVAs were performed. Results show significantly higher (worse) scores in all WRAADDS ED and DERS subscales and significantly lower (worse) scores in all HRQoL scales in the ADHD group (see Table 3), with overall effects for WRAADDS ED [F (3,127) = 23.434, *p* = 0.000, η^2^ = 0.356], DERS [F (6,124) = 6.235, *p* = 0.000, η^2^ = 0.232], and HRQoL [F (4,126) = 13.048, *p* = 0.000, η^2^ = 0.293]. The WRAADDS ED scales *temper control* and *over reactivity* showed a large effect size (η^2^ = 0.155–150) and the *affective lability* scale showed the largest effect size (η^2^ = 0.324). Among the DERS scales, *awareness* showed a small effect size (η^2^ = 0.033), *clarity, non-acceptance, impulse*, and *strategies* showed a medium effect size (η^2^ = 0.066-0.133), and *goals* showed a large effect size (η^2^ = 0.249). Finally, the effect size of the HRQoL scales were small for *life outlook* (η^2^ = 0.053), medium for *relationships* and *psychological health* (η^2^ = 0.097-0.106), and large for *life productivity* (η^2^ = 0.288).

**Table 3:** Group differences on ED and HRQoL scales and subscales

	ADHD	Control	F	η^2^
	M (SD)	M (SD)		
*DERS*				
Non acceptance	14.52 (5.99)	11.20 (3.96)	14.103***	.113
Goals	16.22 (4.47)	12.35 (3.44)	34.972***	.249
Impulse	12.05 (5.73)	9.16 (3.09)	15.476***	.122
Awareness	15.59 (4.41)	13.88 (3.92)	4.378**	.033
Strategies	18.52 (7.61)	13.94 (4.66)	19.577***	.133
Clarity	11.37 (4.60)	9.47 (3.01)	8.981**	.066
Total	89.28 (26.30)	69.38 (14.06)	29.78***	.190
*WRAADDS ED*				
Temper control	1.23 (1.16)	0.45 (0.68)	23.289***	.155
Affective lability	2.07 (0.92)	1.07 (0.57)	60.947***	.324
Over reactivity	1.79 (1.21)	0.99 (0.79)	22.368***	.150
Total sum	19.22 (10.62)	9.28 (6.05)	44.05***	.258
	1.75 (0.97)	0.84 (0.55)		
*HRQoL*				
Life productivity	55.18 (18.92)	76.75 (13.94)	52.201***	.288
Life outlook	60.87 (17.06)	68.94 (13.93)	7.267**	.053
Psychological health	56.26 (23.65)	69.38 (17.40)	15.225***	.106
Relationships	63.97 (19.60)	75.15 (13.28)	13.926***	.097
Total	58.38 (16.18)	73.30 (15.70)	37.21***	.227

*M* = mean; *SD* = standard deviation; DERS = Difficulties in Emotion Regulation Scale total score; WRAADDS ED = Emotional Dysregulation subscales derived from the Wender–Reimherr Adult Attention Deficit Disorder Rating Scale; HRQoL = The adult ADHD quality of life questionnaire total score

*p < .05; **p < .01; ***p < .001

### Moderating role of ED

**Fig. 1:**
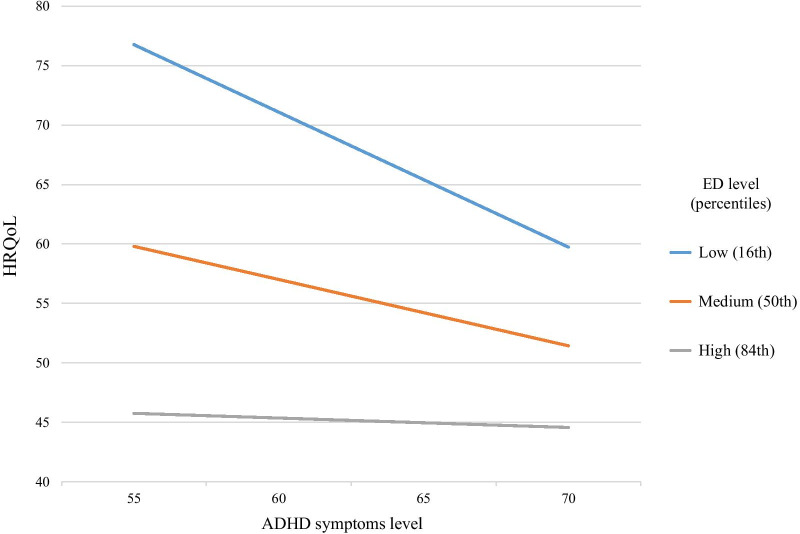
Moderation effect of ED on the relationship between ADHD symptoms and HRQoL. Note. Regression lines show the association between ADHD symptoms and HRQoL for participants with three different levels of ED (low, medium and high). Participants with low levels of ED have higher HRQoL and stronger association between ADHD symptoms and HRQoL. Participants with higher levels of ED report lower HRQoL; for them, there is no association between ADHD symptoms and HRQoL. Note that higher HRQoL scores indicate better HRQoL; higher ED scores indicate worse emotional regulation; higher scores on the ADHD symptoms scale indicate higher levels of ADHD symptoms

To test the hypothesis that ED moderates the relationship between ADHD symptoms and HRQoL for individuals with ADHD, we used moderating analysis of the PROCESS macro (model 1, 5,000 bootstrap resamples) by Hayes (2013). The outcome variable for the analysis was HRQoL. The predictor variable ASRS total score. The moderator variable evaluated for the analysis was WRAADDS ED total score. We found a significant interaction (B = 0.045, SE = 0.022, t = 2.076, *p* = 0.042) between ASRS total score and WRAADDS ED total score. The results identify ED as a negative moderator in the relationship between ADHD symptoms and HRQoL (AAQoL total score) (see Table 4). As can be seen in Fig. 1, as the level of ED gets higher, the relationship between ADHD symptoms and HRQoL is weakened. The interaction was significant also for the DERS as a measure of ED (B = 0.027, SE = 0.009, t = 2.958, *p* = 0.004). Levene’s test of Homogeneity of Variances showed no differences in the variance across the 3 levels of ED (F(2,60) = 1.190, *p* = 0.311), ruling out insufficient variability in the high ED group as a potential account for the results. In addition, there was no floor effect in the AAQoL scores.

**Table 4:** Moderation model

Predictors	Dependent variable = HRQoL				
	β	B	SE	t	*P*
ASRS	− .245	− .607	.230	− 2.630	.011*
WRAADDS ED	− .652	− .998	.145	− 6.884	.000***
ASRS × WRAADDS ED	.188	.045	.022	2.076	.042*

B = unstandardized coefficients; SE = Standard Error; ASRS = adult ADHD Self Report Scale total score; WRAADDS ED = Emotional Dysregulation total score derived from the Wender–Reimherr Adult Attention Deficit Disorder Rating Scale; HRQoL = The adult ADHD quality of life questionnaire total score

## Discussion

The present study had two main goals: to assess the severity of ED among young adults with and without ADHD, and to measure the potential moderating role of ED on the association between ADHD symptoms and HRQoL in young adults with ADHD. Our results show that young adults with ADHD are twice more likely to suffer from ED and that their HRQoL is significantly worse than that of matched non-ADHD controls. In addition, for the ADHD group, ED significantly moderated the relationship between ADHD symptoms and HRQoL, suggesting that high ED levels can overshadow the impact that ADHD symptoms have on HRQoL. These findings support the centrality of ED in ADHD and their crucial influence on everyday HRQoL.

Our study provides novel findings regarding the likelihood of young adults with ADHD to experience ED. The high prevalence of self-reported ED found in our sample (73%) is noteworthy, particularly given the non-clinical nature of the sample. Thus far, clinic-based studies have reported a wide range (34%-86%) of ED in adults with ADHD [27–32]. A recent study conducted in a clinical sample of adults with ADHD (aged 18–75) reported a similar prevalence of ED as was found in our study (70.5%) using the WRAADDS scale [29]. The disparity between the lower expected prevalence in a non-clinical sample and the actual high prevalence that was found, might be attributed to the younger age of our sample. Indeed, younger age was associated with higher ED severity on the sub-scales of temper control and affective lability in a recent study on adults with ADHD using the WRAADDS-SR scale [34]. The prominence of ED in young adults with ADHD in the current study adds to the existing literature which favors the inclusion of ED as a central dimension in ADHD [2, 27, 35–37, 44, 47].

The significant impairments in all domains of HRQoL (productivity, life outlook, psychological health and relationships) in ADHD are in line with the large body of literature emphasizing the negative impact of ADHD on daily health outcomes [2, 5, 14, 15]. So far, studies on adults with ADHD focused mainly on the adverse impact that ED has on functional impairments in ADHD, which goes well beyond ADHD symptoms [27, 36, 44]. In contrast to the measurement of functional impairment, which measures individual performance parameters in a specific task or context, such as work or driving performance, HRQoL assessment allows for a wider perspective on productivity, relationships and well-being. However, only two studies to date reported significant associations between HRQoL in ADHD and a subset of ED constructs, namely emotional impulsivity and deficient emotional self-regulation [44, 47]. The current study adds an additional component by showing the unique contribution of multiple ED aspects to HRQoL, beyond the contribution of ADHD symptoms.

The significant moderating role of ED as measured by both an ADHD-specific tool and a general transdiagnostic tool provides a broader perspective of the impact of different aspects of ED on HRQoL in young adults with ADHD. The impact of the basic characteristics of the emotional experience and expression of ED, specifically in ADHD as reflected by the WRAADDS ED subscales on HRQoL, should be noted. The frequent shifts between emotional states, the disproportional over-reactivity to daily triggers, and the lack of control in face of intense negative emotions causes constant unpredicted interferences in daily life. In addition, the resulted sense of lack of control and coherence regarding the ability of an individual to react as expected might explain the harmful impact of ED on all aspects of HRQoL. The DERS, focusing on the cognitive and behavioral responses to negative emotional states may further explain the negative impact of ED on HRQoL. The difficulties in monitoring one’s emotional state might obscure the identification of the need to detect and implement effective regulatory strategies and control the reaction to negative stimuli. All of these taken together might explain the severe impact of ED on goal-directed behavior. This in turn, resonates with theories of ADHD emphasizing inherent contribution of ED to the executive failure, suggesting constructs such as emotional impulsivity as another core symptom of the disorder [33, 35]. Moreover, the non-acceptance of one’s emotional state may point to a secondary mechanism which further exacerbates of the impact of ED on HRQoL, by impeding an adaptive coping process. This notion is supported by a moderating role of non-acceptance that was found in the relationship between ADHD, depressive symptoms and suicidal ideation [60]. To conclude, the significant moderating role of ED found in both DERS and WRAADDS ED represents the joint contribution of multiple emotional, cognitive, and behavioral ED aspects to the impaired HRQoL in young adults with ADHD.

The broader perspective on ED in the current study can also be discussed in light of the question whether there are facets of ED that are uniquely associated with ADHD [25, 33, 35, 37, 61]. Here, we used two different tools to assess ED in ADHD: an ADHD-specific tool (WRAADDS ED) and a general transdiagnostic tool (DERS) and show substantial ED in ADHD compared to controls using both scales. However, a slightly different picture emerges for the unique ED deficits characterizing ADHD from each tool. For the ADHD-specific ED scale, the subscales of temper control, affective lability and emotional over-reactivity have all yielded large effect sizes for the between-group difference. These findings are consistent with a recent meta-analysis which found large effect sizes for ED severity of ADHD vs. controls [62], specifically in the domains of temper control, affective lability, emotional over reactivity, emotional impulsivity and deficient emotional self-regulation [33–37, 47]. In comparison, the more general, transdiagnostic measure of ED (DERS) yielded a more varied picture, wherein only the *goals* subscale showed a large effect size for group differences; the *awareness* subscale showed a small effect size and the remaining subscales of *clarity, non-acceptance, impulse*, and *strategies* showed medium effect sizes. These findings may reflect a unique profile of ED in young adults with ADHD. Yet, they require further replication and examination in a larger sample, testing whether there are facets of ED that are uniquely associated with ADHD.

The debate regarding the specificity of ED to ADHD vs its transdiagnostic nature could also be addressed by our findings of significant interaction between ED and ADHD symptoms in explaining HRQoL. Our findings show that the strength of association between ADHD symptoms and HRQoL depends on the severity of ED. Thus, higher levels of ED account for the reduced HRQoL seen in individuals who present lower levels of ADHD symptoms. The high prevalence of ED in the ADHD group and the large effect size compared to the control group might support the positioning of ED as a central core symptom in ADHD [29, 33, 35]. Yet, the different pattern of clinical outcomes that depends on ED severity in young adults with ADHD might support the notion that those that have both ADHD *and* high levels of ED form a distinct entity or sub-type [37, 47, 63].

The results of our study have a few clinical implications which underscore the need for the inclusion of a broader assessment addressing ED (among other impairing factors associated with ADHD) [26] and HRQoL in adults with ADHD. Neglecting the potentially harmful impact of ED on the well-being of young adults with ADHD in clinical practice may lead to an inaccurate appraisal of the global clinical severity among young adults. Moreover, our examination of the medication status as a potential confound had no significant effect on the level of ADHD symptoms, ED or HRQoL. In recent placebo-controlled trials, medications status had been shown to effectively improve ADHD core symptoms [64]. ED [65, 66] and HRQoL had also improved though to a lesser extent [67]. Our finding are in line with previous studies which controlled the impact of medication status on ADHD symptoms, ED and HRQoL showing no major differences in outcomes among medicated and non-medicated patients [44, 47]. This understanding, in turn, should encourage further research targeting the underlying mechanisms of ED and the development of effective interventions, accompanying pharmacological treatments, aimed to improve ED and HRQoL among individuals with ADHD. Specifically, the combination of inclusion of self-compassion might serve as a protective mechanism from the possible negative effect of heightened awareness and non-acceptance, reducing the secondary effect of ED on HRQoL [68–71].

Despite its strengths, which included the use of multiple ED assessments and the non-referred sample of young adults with and without ADHD, in contrast to the majority of studies in this field which sample participants from clinics, our study had a few limitations that should be noted. First, the ADHD diagnosis was based on a self-reported valid medical diagnosis of ADHD confirmed by ASRS scores above clinical cutoff, rather than on a clinical interview based on DSM-5 criteria. In addition, our study included only self-report measures of ED and HRQoL, whereas more objective measures might have yielded different outcomes and should be considered in future studies. Finally, studies with larger samples should be conducted in order to confirm and elaborate the results reported here.

## Conclusion

Our results point to the high prevalence of ED in non-referred young adults with ADHD, and to the importance of ED in moderating the association between ADHD symptoms and HRQoL. Thus, high levels of ED explain the significant burden on HRQoL in ADHD beyond symptomatology. Our study is consistent with the literature which found a more severe clinical picture in adults with ADHD who also have high levels of ED [37, 47, 63]. These results stress the significance of ED in everyday life of those struggling with ADHD and call for a more systematic evaluation of ED in the ADHD population.

## Abbreviations

HRQoL: Health Related Quality of Life
ADHD: Attention Deficit Hyperactivity Disorder
ED: Emotion dysregulation
ASRS: Adult ADHD Self-Report Scale
SR-WRAADDS: Self-Report Wender–Reimherr Adult Attention Deficit Disorder Scale
DERS: The Difficulties in Emotion Regulation Scale
AAQoL: The Adult ADHD Quality-of-Life scale
